# Low BRAF V600 mutation prevalence in primary skin nodular melanoma in Indonesia: a real-time PCR detection among Javanese patients

**DOI:** 10.1186/s12919-019-0175-8

**Published:** 2019-12-16

**Authors:** Hanggoro Tri Rinonce, Rovi Panji Mustiko Aji, Ni’mah Hayati, Maria Fransiska Pudjohartono, Bidari Kameswari, Irianiwati

**Affiliations:** 1 Department of Anatomical Pathology, Faculty of Medicine, Public Health, and Nursing, Universitas Gadjah Mada/ Dr. Sardjito Hospital, Radiopoetro Building, 4th Floor, Farmako Street, Sekip Utara, Sinduadi, Mlati, Sleman, Yogyakarta, 55281 Indonesia; 2 Department of Anatomical Pathology, dr. Soeradji Tirtonegoro Hospital, Klaten, Central Java 57424 Indonesia

**Keywords:** Nodular melanoma, Skin tumor, BRAF, BRAF V600 mutation, Indonesia

## Abstract

**Background:**

Cutaneous melanoma is a rare, aggressive skin malignancy with a high mortality rate. Although only contributing 7.6% of the cases worldwide, Asia is responsible for 18.6% of deaths from cutaneous melanoma. BRAF V600 mutation presents a potential prognostic predictor in melanoma. Unfortunately, studies on that mutation in melanoma, particularly nodular subtype, in Indonesia are still scarce. This research aimed to investigate the prevalence of BRAF V600 mutation in primary skin nodular melanoma in Yogyakarta and Central Java, Indonesia. Its association with clinicopathological parameters was also analyzed.

**Methods:**

Forty paraffin-embedded tissue samples from primary skin nodular melanoma cases in 2011–2018 were collected from the two biggest referral hospitals in Yogyakarta and Central Java, Indonesia. The BRAF V600 mutation status was assessed using qualitative real-time PCR and its associations with age, sex, anatomic location, lymph node metastasis, tumor thickness, ulceration, mitotic index, necrosis, lymphovascular invasion, and tumor-infiltrating lymphocytes were analyzed.

**Results:**

BRAF V600 mutations were found in 4 (10%) samples. These mutations were significantly associated with the central (non-extremity) region (*p* = 0.013) and presence of lymphovascular invasion (*p* = 0.005). However, it was not associated with any other variables analyzed in this study.

**Conclusion:**

The prevalence of BRAF V600 mutation in Indonesian primary skin nodular melanoma cases is low and significantly associated with anatomic location and lymphovascular invasion. It is lower than prevalences in other Asian populations as well as in Caucasian populations and suggests that melanoma cases in Javanese people may have distinct clinicopathological characteristics from other Asian ethnicities.

## Background

Cutaneous melanoma is a rare, aggressive skin malignancy with a high mortality rate. The incidence varies among countries, with lower incidence in Asian populations compared to Caucasian populations. Estimates report 0.43–0.48 new cases per 100.000 people in East and South-East Asia, as compared to 12.6–18.8 new cases per 100.000 people in North America and Europe annually [1]. Although only contributing 7.6% to the total global incidence, Asia is responsible for 18.6% of the world mortality from melanoma. This shows that despite its low incidence, melanoma cases in Asia have poor prognoses and are often fatal.

Various clinicopathologic factors affect the outcomes of melanoma patients. Patients with younger age, female gender, extremity location, and no nodal nor distant metastases tend to have better prognoses [2]. Histopathologic factors, such as tumor subtype, thickness, ulceration, mitotic index, lymphovascular invasion, and tumor-infiltrating lymphocytes (TILs), also determine prognosis. Certain genetic mutations can affect melanoma patients’ prognoses, such as the BRAF gene mutation.

The BRAF gene mutation is known as one of the most common mutations in melanoma, with V600 as the most common site of mutation [3]. The discovery of the BRAF V600 mutation opened opportunities for new modes of treatment and prognostic prediction. This prognostic role can be studied through the association between this mutation and the previously identified clinicopathologic factors. However, current evidence on the association between the BRAF mutation and clinicopathologic factors is still sparse and conflicting [2].

The application of these researches to Asian populations is further complicated by the distinct behavior of melanoma in different races. Previous studies suggest that melanoma among Asian patients have different clinicopathologic characteristics from Caucasian patients, especially in subtype frequencies, risk factors, and mutation patterns [4]. The BRAF V600 mutation is found in 40–60% Caucasian patients [3], as opposed to 11.9 to 41.8% Asian patients [5–9]. The current bulk of evidence on BRAF V600 mutations has been obtained from Caucasian patients, deeming it possibly unsuitable for Asian populations.

Indonesia is no exception in this respect, as it also suffers from under-reporting and lack of data on melanoma cases. Due to the scarcity of data, even the most common subtype of melanoma in Indonesia is still questionable. Three different studies reported different subtypes as the most common subtype. One study reported acral lentiginous melanoma as the most common subtype [10], while the Global Burden of Disease study found the superficial spreading subtype [4]. Yet a recent study reported nodular melanoma as the predominant subtype in Yogyakarta, Indonesia [11]. These contradictory results emphasize the lack of data on melanoma in Indonesia. Regarding BRAF mutation studies, only one article has studied the BRAF V600 mutation prevalence among acral lentiginous melanoma in Indonesia so far [12]. No mutation studies have been done on nodular melanoma in Indonesia at all.

Being a relatively uncommon subtype in Asia, nodular melanoma has not been studied much in Asian populations. Despite its low frequency, nodular melanoma is an important contributor to melanoma deaths. A study from Australia reported that although nodular melanomas represented 14% of the invasive melanomas, they were responsible for 43% of the deaths [13]. This dire prognosis further reinforces the necessity for research on nodular melanomas.

Given the lack of evidence for Indonesian populations, further research is needed to elucidate the prevalence of the BRAF V600 mutation and its associations with clinicopathologic parameters among nodular melanoma cases in Indonesia. This research aimed to investigate the prevalence of BRAF V600 mutation in primary skin nodular melanoma in Yogyakarta and Central Java, Indonesia. Its associations with clinicopathological parameters were also analyzed.

## Materials and methods

This retrospective cross-sectional study was conducted in the Department of Anatomical Pathology Dr. Sardjito Hospital, Yogyakarta and dr. Soeradji Tirtonegoro Hospital, Central Java, Indonesia. Both hospitals were the biggest referral hospitals in Yogyakarta Province and Central Java Province located in Java Island, Indonesia. Forty paraffin-embedded tissue samples from primary skin nodular melanoma cases in 2011–2018 were collected and analyzed. All melanoma patients were Javanese, one of the ethnic groups in Indonesia.

The presence of BRAF V600 mutation was assessed using qualitative real-time PCR. Four slices (5 μm thick) of formalin-fixed paraffin-embedded (FFPE) tumor tissues were used for the source of DNA. After deparaffinization and hematoxylin-eosin staining, slides were observed under a microscope and the tumor-containing areas were scraped into tubes for DNA extraction. The DNA extraction was done using the GeneAll® ExgeneTM DNA Extraction Kit (GeneAll Biotechnology, Seoul, Korea) according to the protocol provided by the producer. The obtained DNA was amplified through real-time PCR using the AmoyDx® BRAF V600 Mutations Detection Kit (AmoyDx, Xiamen, Cina). This kit may detect all BRAF V600 mutation types including V600E, V600K, V600D, and V600R.

Clinicopathologic data were obtained from registry records at the Department of Anatomical Pathology Dr. Sardjito and dr. Soeradji Tirtonegoro Hospitals. The data collected consisted of age, sex, anatomic location, lymph node metastasis, tumor thickness, ulceration, mitotic index, necrosis, lymphovascular invasion, and TILs. Anatomic location was classified into extremity and central (trunk, head, and neck) location.

Hematoxylin-eosin stained slides were observed microscopically for lymph node metastasis, tumor thickness, ulceration, necrosis, lymphovascular invasion, and TILs. Presence or absence of lymph node metastasis was assessed by examining lymph node biopsy specimens for tumor cells. Tumor thickness was measured from the granular layer to the deepest level of the tumor, and subsequently classified as ≤4 mm or > 4 mm. Presence of ulceration was defined as thinning of the epidermis to full-thickness epidermal defect. Necrosis was classified into present or absent, with presence of necrosis defined as the presence of an area of necrotic cells covering at least ¼ high power field (0.07 mm^2^). Finding tumor cells identical to the cutaneous melanoma cells in lymph and or blood vessels surrounding the tumor was categorized as the presence of lymphovascular invasion. TILs were defined as lymphocytes migrating from the blood vessels to the peritumoral and intratumoral stroma [14] and classified into absent or present which comprised the brisk and non-brisk category.

For the immunohistochemistry study, paraffin blocks were sliced as thick as 5 μm, deparaffinized, and rehydrated. Subsequently, antigen retrieval was performed using Ventana Ultra Cell Conditioner 1 solution (Ventana Medical Systems, Tucson, AZ, USA) under pH 8–9 in 64 min on 95 °C. Slides were incubated in 3% hydrogen peroxide for 5 min, diluted primary antibody for 30 min, labeled polymer*,* HRP for 30 min, diaminobenzidine for 5 min, and counterstained using hematoxylin for 15 min. Incubation was performed in room temperature. Within the incubation process, slides were washed by tris-buffered saline. Slides were covered by a coverslip. Antibody used in this study was monoclonal Ki67 antibody (Abcam, Cambridge, MA, USA). The mitotic index was calculated as the percentage of positively stained nuclei per 1000 tumor cells and further classified as < 20% and ≥ 20%.

The association between BRAF mutation status and clinicopathologic parameters (age, sex, anatomic location, lymph node metastasis, tumor thickness, ulceration, mitotic index, necrosis, lymphovascular invasion, tumor-infiltrating lymphocytes) was analyzed by the Fisher’s exact test for categorical variables, and the Mann–Whitney test for continuous variables.

## Results

The patients’ age ranged from 21 to 80 years, with an average of 62.35 years of age. Sixteen (40%) patients were male and twenty-four (60%) patients were female. Thirty-three patients (82.5%) had tumors on extremities, while 7 (17.5%) had tumors on the trunk or head and neck (centrally located). Out of the forty samples, BRAF V600 mutations were found in 4 (10%) samples. Among them, three had central lesions while only one had the lesion on an extremity. The association between mutation status and the clinicopathologic parameters is shown in Table 1.

**Table 1:** The association between BRAF mutation status and clinicopathologic parameters

	BRAF (+)	BRAF (−)	*p* value^b^
Age, mean ± SD^a^	56.25 ± 24.55	63.03 ± 12.03	0.926
Age category, n (%)			
< 65 years	3 (7.5)	23 (57.5)	1.000
> 65 years	1 (2.5)	13 (32.5)	
Sex, n (%)			
Male	2 (5.0)	14 (35.0)	1.000
Female	2 (5.0)	22 (55.0)	
Anatomic location, n (%)			
Extremity	1 (2.5)	32 (80.0)	**0.013**
Central	3 (7.5)	4 (10.0)	
Lymph node metastasis, n (%)			
Present	2 (5.0)	15 (37.5)	1.000
Absent	2 (5.0)	21 (52.5)	
Tumor thickness, n (%)			
≤ 4 mm	1 (2.5)	5 (12.5)	0.493
> 4 mm	3 (7.5)	31 (77.5)	
Ulceration, n (%)			
Present	2 (5.0)	21 (52.5)	1.000
Absent	2 (5.0)	15 (37.5)	
Mitotic index, mean ± SD^a^	28.25 ± 17.60	22.11 ± 17.67	0.487
Mitotic index category, n (%)			
≥ 20%	3 (7.5)	16 (40.0)	0.331
< 20%	1 (2.5)	20 (50.0)	
Necrosis, n (%)			
Present	2 (5.0)	26 (65.0)	0.570
Absent	2 (5.0)	10 (25.0)	
Lymphovascular invasion, n (%)			
Present	4 (10.0)	8 (20.0)	**0.005**
Absent	0 (0.0)	28 (70.0)	
Tumor-infiltrating lymphocytes, n (%)			
Present	4 (10.0)	24 (60.0)	0.297
Absent	0 (0.0)	12 (30.0)	

^a^SD = standard deviation

^b^*P* value < 0.05 was considered significant. Significant values are indicated in bold

Positive BRAF V600 mutation was significantly associated with central anatomic location (*p* = 0.013) and lymphovascular invasion (*p* = 0.005). There were no significant associations between BRAF mutation status and age, sex, lymph node metastasis, tumor thickness, ulceration, mitotic index, necrosis, nor tumor-infiltrating lymphocytes.

## Discussion

In this study, we studied the prevalence of BRAF V600 mutation in primary skin nodular melanoma and its association with clinicopathological parameters. The BRAF V600 mutation was found in 4 patients, yielding a prevalence of 10% among nodular melanoma cases. There is little comparable data in Asia due to the limited studies on nodular melanomas. A study in Japan reported that 50% of nodular melanoma cases had the BRAF V600 mutation [6], while another research in Turkey stated the percentage at 29.4% [15].

When compared to the previous studies for all melanoma subtypes in Asia, the prevalence of BRAF V600 mutation in this study was still lower than other Asian countries. Asian melanoma cases are dominated by the acral lentiginous subtype [4], while nodular melanoma was the most common subtype in Yogyakarta and Central Java, Indonesia [11]. This subtype pattern resembled two studies, in Mexico and Germany respectively, which also found nodular melanoma as the most common subtype [16, 17]. Nodular melanomas are known to have BRAF V600 mutation rates approximately twice higher than acral lentiginous melanomas [18]. However, the prevalence found in this study was lower than the previous findings for all melanoma subtypes in Asia (11.9 to 41.8%). This outcome further reaffirms that Indonesian melanoma cases do have a significantly lower BRAF V600 prevalence compared to Asia in general.

Researches from nodular melanoma cases outside Asia reported higher prevalence results compared to this study. Studies from Caucasian populations, such as Australia and the United States, Germany, and Norway, report prevalence of BRAF V600 mutations in nodular melanomas at 22.37% [19], 37.7% [17], and 40.84% [20], respectively. A study in Mexico reported the prevalence of the mutation at 29.09% in nodular melanomas [16]. In Brazil, 80% nodular melanomas had the BRAF V600 mutation [21]. A Nigerian study yielded a prevalence of 11% in all melanoma samples [22], a closer number to our results. However, the detection of BRAF V600 mutation in this Nigerian study was done using immunohistochemistry, which is less sensitive compared to PCR studies. Hence, the prevalence when measured using the PCR technique would most likely be higher still in African populations.

The unusual subtype distribution and mutation pattern may indicate that melanoma cases in Javanese ethnic indeed present an anomaly among other Asian populations. Genetic variations between the different ethnicities may influence the prevalence of certain mutations [23], which may explain the wide variation of BRAF V600 mutation rates in Asia. The current evidence from Asia mostly studied East Asia populations, with nearly no data from South-East Asia regions. Melanomas with wild-type BRAF likely have mutations in the upstream proteins of the MAPK pathway, such as NRAS or KIT [24]. The low BRAF mutations prevalence in this study should prompt further studies to investigate upstream mutations in the MAPK pathway in South-East Asia, including Indonesia.

Unique combinations of underlying pathophysiological factors may also affect the BRAF V600 mutation prevalence. Sun exposure is one of the best-known factors in melanoma pathogenesis. Ultraviolet radiation can induce damage to DNA, including the BRAF gene [24]. However, BRAF mutation more frequently appears in melanomas in locations without chronic sun damage. Melanoma cases in Asian patients tend to appear in areas that are rarely sun-exposed, giving rise to the conjecture that sun exposure does not play a major role in Asian melanomas [4]. In our study, 33 out of 40 patients had lesions on the extremities which were more exposed to the sun compared to central locations. Considering that central locations are associated with BRAF mutations both in previous studies [18] and this study, the low percentage of centrally located lesions may contribute to the lower prevalence of the BRAF V600 mutation.

Differences in population characteristics and research methods may also have contributed to the lower prevalence of BRAF V600 mutation. The older population in this study (with 65% participants above 65 years of age) may have influenced the mutation prevalence, as BRAF V600 mutation is associated with younger patients [25]. More advanced methods, such as next-generation sequencing, would have enhanced the sensitivity of BRAF V600 mutation detection [26] and enabled more in-depth study of specific mutation types (such as V600E, V600K, V600D, and V600R) [27].

BRAF V600 mutation showed associations with clinicopathologic characteristics, namely central location and the presence of lymphovascular invasion. Previous melanoma studies report that BRAF V600 mutations were found approximately twice more often in the trunk compared to non-trunk locations [18]. Central (trunk, head, and neck) areas receive less sun exposure and chronic sun damage, which is associated with higher rates of BRAF mutation in various studies. A study in Boston also reported higher rates of lymphovascular invasion with BRAF mutation [28]. Lymphovascular invasion happens earlier and is a more sensitive examination than lymph node metastasis, hence explaining the association with lymphovascular invasion but not lymph node metastasis. No significant associations were found between the BRAF V600 mutation with age, sex, lymph node metastasis, tumor thickness, ulceration, mitotic index, necrosis, and presence of tumor-infiltrating lymphocytes. These findings mirror most of the findings from previous studies in Asia.

Neither BRAF mutation testing nor its inhibitor has been widely used in clinical management of melanoma cases in Indonesia. Up to now, our national health insurance program does not yet cover BRAF inhibitor as the therapy for melanoma. The patients were mostly treated by decarbazine. Therefore, there is no published study about the efficacy of BRAF inhibitors in Indonesian populations. Further studies are needed to address this issue. This study will become a foundation for pioneering the application of BRAF mutation detection for prognosis and therapy purposes in Indonesia. Information on the patients’ mutation status can help predict the survival rates. The patients identified with the mutation may get benefits from BRAF inhibitor therapy. With the known clinicopathologic associations with this mutation, we can be more selective in which patients would likely benefit from BRAF mutation testing and inhibitor therapy. With further research, BRAF mutation testing and therapy can be more widely implemented in South-East Asia, especially Indonesia.

## Conclusion

BRAF V600 mutations were found in 10% of primary skin nodular melanoma cases in Yogyakarta and Central Java, lower than previous studies both among Asian and Caucasian populations. This low mutation prevalence and the unusual nodular subtype predominance suggest that melanoma among Javanese ethnic may have distinct clinicopathological characteristics from other Asian ethnicities. The presence of the BRAF V600 mutation is significantly associated with anatomic location and lymphovascular invasion. There is a need for further research using more advanced methods (such as next-generation sequencing), specifying the subtype of the BRAF V600 mutations, and investigating other mutations in the MAPK pathway, such as NRAS or KIT, in South East Asia, including Indonesia.

## Data Availability

All data generated or analyzed during this study are included in the submission. The raw data are available from the corresponding author on reasonable request.

## Abbreviations

DNA: Deoxyribonucleic acid
FFPE: Formalin-fixed paraffin-embedded
HRP: Horseradish peroxidase
MAPK: Mitogen-activated protein kinase
PCR: Polymerase chain reaction
TILs: Tumor-infiltrating lymphocytes
